# Cohesive and Non-Cohesive Gold

**Published:** 1875-02

**Authors:** J. S. Rice

**Affiliations:** Shelbyville, Indiana


### COHESIVE AND NON-COHESIVE GOLD.

BY J. S. RICE, M. D., SHELBYVILLE, INDIANA.

*An Essay read before the Eastern Indiana Dental Association.*

*Mr. President and Gentlemen:*

Much has been said lately of the comparative merits of cohesive and non-cohesive gold as a dental stopping, and although this agitation does not emanate from gentlemen who have distinguished themselves as workers of cohesive foil; yet men, not unknown to fame in the dental profession, have not only called into question the entire system of contour filling, but have declared the whole process of filling with cohesive gold a failure; and by recital of ingenious experiments, and theories of no little plausibility, they endeavor to explain to us that contour fillings can not stand as a house built upon a rock, but that the waters rush about them and, under them, and they vascillate, and totter, and are gone. If we accept these views, the efforts of the majority of the most skillful operators in the country have not only been wasted during several years, but the public confidence which they have inspired, by their zealous assurances, and the beauty of their work, will have been deceived, and the calamity to the profession must necessarily be great, and many disappointed and conscience-stricken dentists must expiate their folly for having basked in this glowing ideal, and exclaiming "Eureka!" when they in reality had not found it. The big file and thick-edged corundum disc must be installed, irregular walls must be leveled, and cylinders and wads of non-cohesive gold piled and propped flush to their borders.

Public taste must be cultivated to appreciate the improved form of bicuspids and molars filled to resemble canines, and the dentist, who must not only be an artist but a philosopher as well, can calm the apprehensions of patients who may think these slopes will cause food to glide and wedge unpleasantly, by explaining that such ideas, though plausible, are practically an illusion, and that the point of the tongue passed occasionally through these spaces not only acts effectually in dislodging any accidental accumulation, but after a little practice becomes a pleasant pastime, besides adding to expression by relieving the monotonous repose of the features.

If we must accept such conclusions as have been formed by some recent writers, the use of amalgam must inevitably increase to the extent of at least a few tons annually, for it is remarkable that the distinguished gentlemen who find cohesive gold so unfit a material for filling teeth, breathe no word of harm to amalgam, and some have found it a good preserver, especially when unwashed and with no surplus mercury squeezed out, and one gentleman particularly, who has been most prominent in pointing out the shortcomings of cohesive foil and contour fillings has himself discovered and placed in the market an amalgam which he knows to be non-shrinkable and good, unless it be exposed to the *absurd and peculiarly American process of washing and squeezing.*

Now is it true that cohesive gold can not be applied to the walls of a cavity so as to thoroughly occupy and hermetically seal it? Can it not be adapted to every variety of surface and margin, and welded into one solid mass and extended to protect exposed borders, and restore form, and at the same time remain unimpared as a protector of the cavity proper? Or do they tell us that these fillings contract and expand keeping up an ebb and flow of fluid between the gold and the wall of the cavity, or is there a catalytic action or a peculiar something else which causes enamel and dentine to perish in the presence of cohesive gold.

No such conjectures have facts to sustain them, we repeat, that this condemnation of contour fillings by distinguished dentists does not come from men who, had become known as experts in the use of cohesive gold; they indeed may have tried it and become dissatisfied as have sculptors endeavored to form from a block of marble a Venus or an Apollo, and failing in their efforts have found plaster of Paris yield a readier obedience, nevertheless marble continued to be used by their contemporaries beneath whose hands it took satisfactory forms.

The opinion of the essayist strengthened by much observation and considerable experience, is that cohesive gold in the hands of an operator who has become expert in its use is for general application to all classes of cavities, the most reliable dental stopping at present known, that it can be adapted to all the inequalities of walls and margins of any ordinary cavity is a certainty, and that it may in many cases be firmly anchored in the main cavity, and built out for the restoration of any desired contour is a property which forms at once its most beautiful and useful attribute.

But to use it successfully absolute thoroughness in the preparation of the cavity, and the manipulation of the filling material are requisite. Where frail walls that can not be protected are left standing, and chalky enamel or softened dentine forms any part of the margin of the cavity, no filling, however perfect, can arrest decay, and should the cavity be faultlessly prepared and be not accurately filled and finished smooth, full and solid to its edges, the operation will surely fail. We think that it is under circumstances of this nature, that unfavorable observations have been made regarding cohesive gold.

Yet as to soft foil, no one who is acquainted with its time-tried qualities can dispute its usefulness, and the dentist who is unacquainted with the manner of manipulating it has omitted an important acquirement; in suitable cavities it can be adapted with great accuracy and protects perfectly so long as exact and entire closure of the cavity is maintained, but the very quality of soft gold that is claimed as its greatest virtue —its non-union—renders it incapable of forming a filling of sufficient tenacity to resist the mechanical forces incident to its use in the mouth, and as it is thus entirely inapplicable to any outward restoration beyond the margins of the cavity, it can not, if used alone, be successfully applied except in deep cavities with good strong walls, Cohesive gold, on the other hand, both preserves and restores, and our assertion that it fills the requirements of a permanent filling material more fully and approaches nearer to our beau ideal of a dental preserver and restorer than any other one substance in use, we think, is corroborated by the experience and observation of scores of the best operators in the world. But to avail ourselves of the advantages of this material, and to be able to use it with success, we must be in possession of a discretion and knowledge of principles which no amount of ingenuity or mechanical genius can replace. Nevertheless skillful manipulation stands prerequisite, the intractable coffer dam must be taught obedience to the fingers, the slabs or pellets of gold must be taught not to roll and crumble, but to take up their position with alacrity before the instrument. Cavities must be cut until thoroughly formed to a retaining shape, and on this point there should not remain a shadow of doubt when preparing an anchorage you may meet in your path a ridge of solid enamel that laughs at steel, but go through it, cut it with a stone, or crumble it with bur or file; retaining points always fall in tender places, and as the patient shrinks before the drill, our sympathies struggle with our judgment, but in this matter we must gently persist till we gain the point. Some patients object to the time and expense necessary for contour work. Many are unable to meet the expense of having it done at all, yet this offers no obstacle to our supplying it to such as appreciate it, and have the currency and the will to compensate us.

